# Impairment of Myocardial Mitochondria in Viral Myocardial Disease and Its Reflective Window in Peripheral Cells

**DOI:** 10.1371/journal.pone.0116239

**Published:** 2014-12-31

**Authors:** Jin Wei, Deng-Feng Gao, Hao Wang, Rui Yan, Zhi-Quan Liu, Zu-Yi Yuan, Jian Liu, Ming-Xia Chen

**Affiliations:** 1 Department of Cardiology, the Second Affiliated Hospital of Xi’an Jiaotong University, Xi’an, Shaanxi, China; 2 Department of Anesthesiology, Wake Forest University School of Medicine, Medical Center Boulevard, Winston-Salem, North Carolina, 27157-1009, United States of America; 3 Department of Cardiology, the First Affiliated Hospital of Xi’an Jiaotong University, Xi’an, Shaanxi, China; 4 Medical College of Xi’an Jiaotong University, Xi’an, Shaanxi, China; Osaka University Graduate School of Medicine, Japan

## Abstract

**Background:**

Viral myocardial disease (VMD) is a common disease inducing heart failure. It has not been clear the roles of mitochondrial damage in the pathological changes of cardiomyocytes in VMD.

**Methods:**

Myocardial tissues and lymphocytes were collected from 83 VMD patients. Control groups included 12 cases of healthy accidental death with myocardial autopsy and 23 healthy blood donors. The mouse model of viral myocarditis (VMC) was established by Coxsackie virus B_3_ infection and myocardial tissues and skeletal muscle were collected. Mitochondrial DNA (mtDNA) deletion rate was quantitatively determined using polymerase chain reaction.

**Results:**

There was significantly difference of myocardial mitochondrial DNA deletion rate between VMD or VMC group and control group (*P*<0.05). Moreover, the loss of mitochondrial membrane phospholipids was significantly different between VMD or VMC group and control group. In VMC mice, there were negative correlations between myocardial mtDNA^3867^ deletion rate and left ventricular peak systolic pressure (LVPSP) (r = −0.66, P<0.05), and between myocardial mtDNA^3867^ deletion rate and +dp/dt_max_ (r = −0.79, P<0.05), while there was positive correlation between myocardial mtDNA^3867^ deletion rate and −dp/dt_max_ (r = 0.80, P<0.05).

**Conclusion:**

Mitochondrial damage is an important pathophysiological mechanism leading to myocardial injury and cardiac dysfunction. The mitochondrial damage in the skeletal muscle and lymphocytes reflect a “window” of myocardial mitochondrial damage.

## Introduction

Viral myocarditis (VMC) is mainly characterized with non-specific myocardial interstitial inflammatory lesions caused by viral infection [1]. Evidence shows that patients with persistent viral infection in myocardium finally develop into dilated cardiomyopathy (DCM) [2], [3]. Therefore, viral myocarditis is also considered the early stage of dilated cardiomyopathy [4]. Both VMC and DCM are generally referred to viral myocardial disease (VMD) [5]–[8].

Mitochondria are the main energy engines in the cells. The abnormality of myocardial energy metabolism is one of the important mechanisms of the development of cardiomyocyte hypertrophy and heart failure [9]–[11]. The existence of multiple deletions and mutations of mitochondrial deoxyribonucleic acid (mtDNA) in patients with dilated cardiomyopathy has been demonstrated by Arbustini and other investigators [12]. But it has been unclear yet if these changes are the cause or the sequence of myocardial injury. The present study was designed to provide more evidence for the roles of the mitochondrial damage in the pathogenesis of viral myocardial disease. Moreover, given the limitations of clinical application of myocardial biopsy, we also studied the mitochondrial damages in skeletal muscle, peripheral blood samples, and myocardium and analyzed their correlations in order to obtain a “window” in peripheral cells which reflects mitochondrial damage in myocardium.

## Materials and Methods

### Viral myocardial disease patients and control group

Data was collected from 83 patients with viral myocardial disease in the inpatients units of the Department of Cardiology, Affiliated Hospital of Xi’an Jiaotong University, collected from January 1997– July 2000. These patients included 50 patients with viral myocarditis (blood coxsackievirus B-IgM was positive for all of these 50 patients) and 33 patients with dilated cardiomyopathy (the clinical data of patients in Table 1). The diagnosis of viral myocarditis was based on the criteria established by the Chinese Heart Association in 1999 [13]. The diagnosis of dilated cardiomyopathy was based on the criteria established by the World Health Organization/International Federation of Society of Cardiology (WHO/ISFC) in 1996 [14]. All the DCM patients included in this study had the history of viral myocarditis. Myocardial biopsy has been performed via right jugular vein catheterization in 20 cases of viral myocarditis and 12 patients of dilated cardiomyopathy. Myocardial tissues were collected at 2–4 sites on the right side of interventricular septum using Cordis biopsy forceps and immediately stored in liquid nitrogen. Lymphocytes were isolated from 10 ml of blood of patients and saved at −80°C for the later analysis. Control groups included 12 cases of healthy accidental death (10 males and 2 females) for the myocardial autopsy in the same sites of myocardium and 23 healthy blood donors (11 males and 12 females) for the normal subject control. Although only 32 out of 83 patients agreed to have cardiac biopsy, we received blood samples from all the cases participated in this study. There were no age differences among groups of total 50 patients of viral myocarditis, 20 patients of viral myocarditis with cardiac biopsy, total 33 patients of dilated cardiomyopathy, 12 patients of dilated cardiomyopathy with biopsy, 12 cases of healthy accidental death, and 23 healthy blood donors. Written consent was obtained from all of the participants or family members of the healthy accidental death in the study and the study protocol was approved by Institutional Review Board of Xi’an Jiaotong University School of Medicine.

**Table 1:** The clinical data of including VMD patients (mean ± SEM).

	Dilated cardiomyopathy	Viral myocarditis
sample size	33	50
Age (years)	43.0±13.2	32.0±11.4
Gender (male/female)	19/14	22/28
Course of disease (months)	19.7±17.5	7.9±18.9
NYHA	3.18±0.77	1.16±0.51
LAD(mm)	38.39±8.44	28.18±4.36
LVEDs (mm)	53.33±14.98	30.52±5.49
LVEDd (mm)	65.09±12.38	48.29±5.19
EF (%)	37.73±18.06	66.31±8.19
CTR	0.63±0.09	0.46±0.06
Arrhythmia		
super ventricular	2	10
ventricular	13	21
atrioventricular block	2	4
intraventricular block	9	0

NYHA, New York Heart Association; LAD, Left Atrial Diameter; LVEDs, Left Ventricular End-Systolic Diameter; LVEDd, Left Ventricular End-Diastolic Diameter; EF, Ejection Fraction; CTR, Cardiothoracic Ratio.

### Viral myocarditis mice model

#### Establishment of the mouse model

Mouse model of viral myocarditis was established as described previously [15]. Forty of 6–8 weeks old male BALB/c mice with body weight of 18–22 g were obtained from Xi’an Jiaotong University School of Medicine Laboratory Animal Center, and were randomly divided into 2 groups. All animal study protocols were approved by the Institutional Animal Research and Ethics Committee of Xi’an Jiaotong University (SCXK2007-001). The experimental group with 30 mice were intraperitoneally injected with 0.1 ml of Eagle solution containing Coxsackie virus B_3_ [CVB_3_, The 50% infectious dose (TCID_50_) = 10^8^] (provided by the laboratory of viral heart disease at Fudan University Zhongshan Hospital). Ten mice in control group were intraperitoneally injected with the equal volume of Eagle medium without CVB_3_. We found that the mice injected with CVB_3_ began to show towering hair, curling, reduced activity, reduced body weight in 3 days after injection, mild systemic cyanosis in 4 days, progressive hind limb paralysis, ear and tail ischemia, necrosis, and scab in 6 days after injection. 3 mice died in 7∼15 days after injection and no more death afterward.

#### Determination of heart function

At day 3, 11, and 24 after viral injection, heart functions were measured using multi-channel polygraph system (Pawerlab, USA) through carotid artery catheterization under the anesthesia of 20% urethane (0.01 ml/g body weight, intraperitoneal injection).

#### Sample collection

Myocardium from heart apex and skeletal muscle from forelimb were collected after the measurement of heart functions. Each tissue was cut into two 1 mm^3^ pieces for the following experiments: the first fixed using improved Demer’s method, serially dehydrated in acetone, embedded in Epson 812 and sectioned with a microtome. The sections were observed directly under a transmission electron microscope without uranium staining [16]. The second homogenized in 1000 µL of Tris-EDTA (TE) buffer. The tissues were digested with 1% trypsin (final concentration was 1–2.5 µg/mL) at 37°C for 24 h. Phenol/Chloroform extraction followed by ethanol precipitation was used to isolate total DNA. The quality and quantity of DNA were measured by ultraviolet spectrophotometry [17].

### Quantitative analysis of mitochondrial DNA deletion

#### Quantitative analysis of mitochondrial DNA^4977^ and mtDNA^7436^ deletion rate of myocardium and lymphocytes in VMD patients by polymerase chain reaction (PCR)

The common mtDNA deletion types, mtDNA^4977^ and mtDNA^7436^ deletion, were compared in VMC and DCM patients. Three primers were needed for PCR analysis [18]–[22]: Primers P1/P2 (located in mtDNA L8282–8305 and H13832–13852) were used to amplify a fragment of 594 base pairs (bp) representing mtDNA^4977^ deletion. Primers P3/P4 (located in mtDNA L8150–8166 and H16142–16159) were used to amplify a fragment of 574 bp representing mtDNA^7436^ deletion. Primers P5/P6 were designed in the highly conserved region of the mtDNA molecule 12S rRNA gene region, located in the L1077–1098 and H1552–1572 position. For either wild type or mtDNA deletion, P5/P6 could amplify a fragment of 496 bp which was used as an internal reference in order to calculate the mtDNA^4977^ and mtDNA^7436^ deletion ratio. The PCR reaction system included 0.4 µg of DNA, 200 µmol/L of 4×dNTP including α-^32^P-dATP (3000 Ci/mmol), 0.5 µmol/L of each primer, MgCl_2_ 2 mmol/L, TaqDNA enzyme (Promega) 2U. The conditions of PCR were as follows: pre-denaturing at 94°C for 7 min, denaturing at 94°C for 60 s, annealing at 55°C for 60 s, extension at 72°C for 105 s, 35 cycles, final extension at 72°C for 8 min. The PCR products from primers P1/P2 and P3/P4 were digested with restriction enzyme DdeI and XbaI to confirm the specificity of the PCR products. For quantitative analysis of PCR products, the PCR products were electrophoresed on a 1.2% agarose gel and observed under ultraviolet light. Specific bands were cut and put in the scintillation vial with 0.4 ml of 30% H_2_O_2_. After the gel was dissolved at 70°C in a water bath, 5 ml of scintillation fluid was added and put in the dark for overnight followed by the liquid scintillation counting. To eliminate the quenching effect of the gel, exactly 10 mg of the gel was cut and used for quench correction. Finally, an amplification curve was made with the number of PCR cycles and the amount of PCR products (cpm number) as the horizontal and vertical axis, respectively. To have the best amplification efficiency, our pilot studies have shown that the best amount of DNA template is 0.4 µg, and the best number of PCR cycles is 35 cycles. The ratio of product from P1/P2 over the product from P5/P6 reflected the rate of mtDNA^4977^ deletion over the total mtDNA. The ratio of product from P3/P4 over the product from P5/P6 reflected the rate of mtDNA^7436^ deletion over the total mtDNA.

#### Quantitative analysis of mitochondrial DNA^3867^ deletion rate (mtDNA^3867^) in myocardium and skeletal muscle of mouse using PCR

DNA^3867^ deletion has been found to be the most common deletion type in the heart of mice [19], [21], [23]. Three primers were needed for the PCR assay (mtDNA sequence of BALB/c mice was referred according to GeneBank NC001569): primer L1 (8858–8877): 5′TCTATTCATCGTCTCGGAAG3′, primer L2 (12885–12904): 5′TACCATTCCTAACAGGGTTC3′, and primer H (13354-13335): 5′TTTATGGGTGTAATGCGGTG3′. With primer L1/H, a DNA fragment of 630 bp was amplified reflecting the deleted-type of mtDNA^3867^. With primer L2/H, a DNA fragment of 476 bp was amplified reflecting the wild-type of mtDNA, which was normal content of mtDNA and was used as internal standard. The PCR reaction system included 0.3 µg of DNA, 0.5 µmol/L of each primer, and 10 µL of deionized water. After pre-denaturing at 95°C for 5 min, the cuvette was immediately put into 4°C of water, then 25 µL of 2×PCR Master (containing MgCl_2_ 1.5 mmol/L, 4×dNTP 200 µmol/L, TaqDNase 0.1 U/µL) was added and the total reaction volume was adjusted to 50 µL with deionized water. PCR reaction conditions were as follows: denaturing at 94°C for 45 s, annealing at 53°C for 45 s, extension at 72°C for 60 s, 30 cycles, final extension at 72°C for 7 min. The amplified products were digested with restriction enzyme Hinf I, electrophoresed on a 1.5% agarose gel and stained with ethidium bromide. Image Master VDS analyzing system was used for identifying the specificity. The ratio of deleted-type of mtDNA^3867^ to wild-type of mtDNA plus deleted-type of mtDNA^3867^ represented the deletion rate of mtDNA^3867^ in the heart.

#### Determination of the loss of mitochondrial membrane phospholipids in lymphocytes in VMD patients

Lymphocytes were fixed using Demer’s method, serially dehydrated in acetone, embedded in Epson 812 and sectioned with LBK. The sections were observed directly under a transmission electron microscope without uranium staining. For lymphocytes samples, 100 mitochondria were randomly selected and the loss rate of mitochondrial membrane phospholipids localization was calculated according to three conditions: inner or external membrane normal localization, partial loss, and most/complete loss.

#### Determination of the loss of mitochondrial membrane phospholipids in myocardium and skeletal muscle

Myocardium and skeletal muscle were cut into 1 mm^3^ pieces, fixed using Demer’s method, serially dehydrated in acetone, embedded in Epson 812 and sectioned with LBK. The sections were observed directly under a transmission electron microscope without uranium staining. For the cardiac samples, the sections were observed at 2×10,000 magnification, 10 photos were randomly selected and the total number of mitochondria was recorded. The loss rate of mitochondrial membrane phospholipid localization was calculated according to three conditions: inner or external membrane normal localization, partial loss, and most/complete loss.

### Statistical analysis

All data are expressed as mean ± SEM. ANOVA analysis was performed using SPSS software, and significant interactions between the groups were further characterized using Student-Newman-Keuls post hoc analyses. All the correlation assays in this study were assessed using the Spearman test. P<0.05 was considered statistically significant.

## Results

### mtDNA deletion in VMD patient and animal model

#### Myocardial mtDNA deletion in VMD patients

Both myocardial mtDNA^4977^ and mtDNA^7436^ deletions existed in all the groups: normal subjects and VMC or DCM patients. However, the deletion rate in normal subjects was very low (0.175%). The total mtDNA deletion rate in VMC group was 0.385%, which were 2.2 folds higher than that in normal group. The total mtDNA deletion rate significantly increased in DCM group (3.004%), 16.2 and 6.8 folds higher than normal subjects and VMC patients respectively (Table 2).

**Table 2:** MtDNA deletion rate in myocardium of VMD patients (mean ± SEM).

mtDNA deletion rate (%)	myocardium						lymphocytes			
	controls	VMC	DCM	F value	P value	controls	VMC	DCM	F value	P value
n	12	20	12			23	20	12		
Age (years)	32.6±11.2	31.4±9.80	39.6±11.0	2.4	0.1	37.7±4.7	31.4±9.8	39.8±11.0	4.7	0.014
MtDNA^4977^ deletion rate (%)	0.11±0.10	0.20±0.09	1.54±1.55	12.7	<0.001	0.02±0.01	0.16±0.08	1.07±1.61	8.5	0.001
MtDNA^7436^ deletion rate (%)	0.06±0.10	0.19±0.09	1.46±1.34	15.8	<0.001	0.01±0.01	0.15±0.06	0.98±1.07	16.2	<0.001
Total mtDNA deletion rate (%)	0.18±0.13	0.39±0.14	3.00±2.85	14.6	<0.001	0.03±0.01	0.31±0.11	2.06±2.66	11.6	<0.001

#### Lymphocytes mtDNA deletion and its correlation with that in myocardium in VMD patients

**Figure 1:**
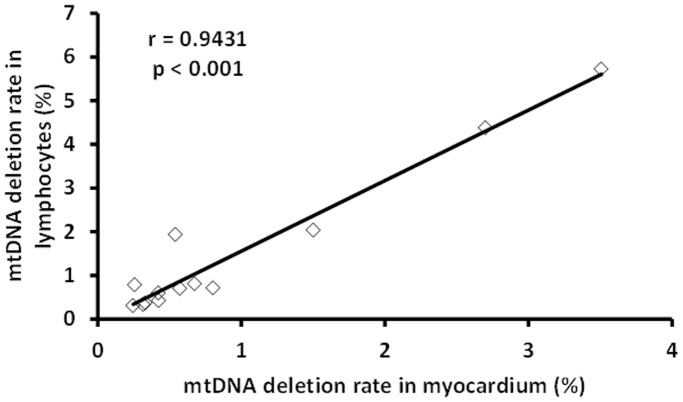
The correlation curve of summation mtDNA deletion rate between myocardium and lymphocytes in VMD patients.

Both mtDNA^4977^ and mtDNA^7436^ deletions existed in lymphocytes of normal subjects, VMC and DCM patients. The mtDNA deletion rate of lymphocytes in VMC patients was significantly increased compared with normal subjects, while it was higher in DCM patients than that in VMC patients (Table 2). Moreover, the patterns and the degrees of the differences of the mtDNA deletion rate in lymphocytes were similar to that in myocardium. Spearman correlation analysis showed that the correlation coefficients between myocardium and lymphocytes were 0.956 (P<0.05), 0.960 (P<0.05), and 0.943 (P<0.05) for mtDNA^4977^ deletion, mtDNA^7436^ deletion, and the total deletion rate, respectively (Fig. 1).

#### Myocardial mtDNA^3867^ deletion in VMC mice

**Figure 2:**
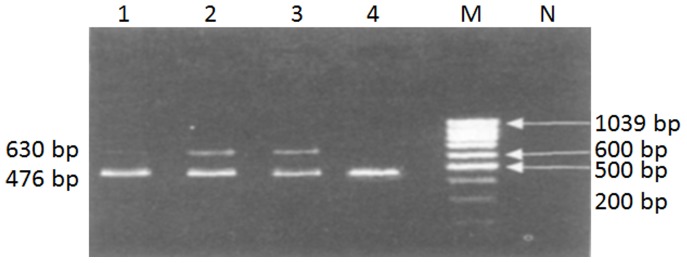
PCR result of myocardial mtDNA in VMC mice. Lane 1: myocardial mtDNA of 3 days after viral injection; lane 2: myocardial mtDNA of 11 days after viral injection; lane 3: myocardial mtDNA of 24 days after viral injection; lane 4: myocardial mtDNA of control mouse. M: DNA marker; N: negative control.

Myocardial mtDNA^3867^ deletion existed in both CVB3 injected and control groups. But the deletion rate in control group was very low (0.00211%). At day 3 after the virus infection, myocardial mtDNA^3867^ deletion rate significantly increased compared with control (*P*<0.05), and increased further at day 11d (*P*<0.05 vs. day 3). At day 24, the deletion rate tendered to be lower but there was no significantly difference compared with day 11 (Table 3, Fig. 2).

**Table 3:** Heart function, and MtDNA^3867^ deletion rate in myocardium and skeletal muscle of VMC mice (mean ± SEM).

Viralinfection days	n	SBp(mmHg)	DBp(mmHg)	LVPSP(mmHg)	+dp/dtmax(mmHg·s-1)	−dt/dt max(mmHg·s-1)	MtDNA^3867^deletionrate in myocardium (%)	MtDNA^3867^deletionrate in skeletal muscle (%)
Controls	10	92.5±8.4	61.3±8.5	99.6±8.2	4903±668	−4173±596	0.0021±0.0003	0.000133±0.00002
3	10	80.5±6.5	56.5±7.6	88.1±3.8	4943±327	−3315±393	0.0197±0.0012*	0.000964±0.00006*
11	10	78.3±4.9*	49.2±4.2*	79.6±4.7*	3088±268*	−2463±360*	0.0329±0.0031* ^#^	0.001390±0.00007* ^#^
24	7	82.6±3.8*	51.8±4. 6	84.9±2.8*	3689±252*	−2934±301*	0.0264±0.0009*	0.00105±0.00006*

*P<0.05 vs. controls; ^#^P<0.05 vs. 3d after viral infection. SBp: systolic blood pressure; DBp: diastolic blood pressure; LVPSP: left ventricular peak systolic pressure; +dp/dtmax: the maximum rate of rise of LVPSP; −dp/dtmax: the maximum rate of fall of LVPSP.

#### mtDNA^3867^ deletion in skeletal muscle and its correlation with that in myocardium in VMC mice

**Figure 3:**
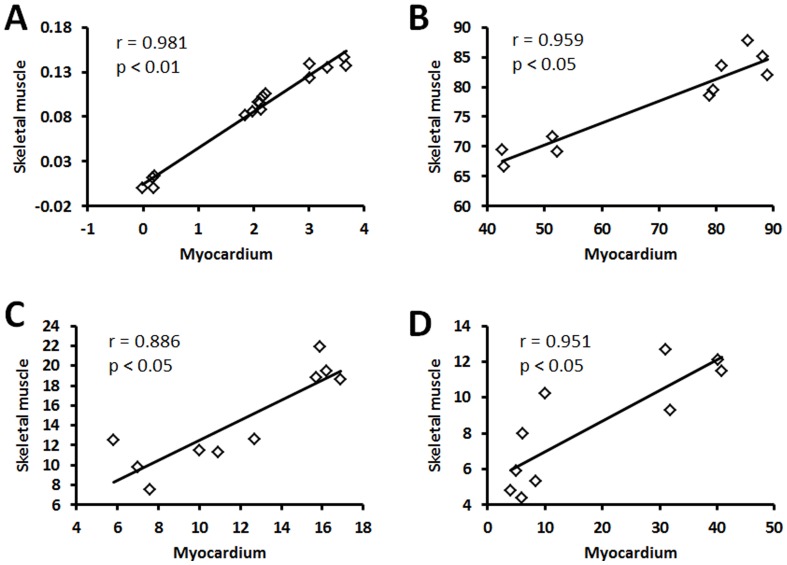
The correlation curves of mtDNA deletion rate between myocardium and skeletal muscle, and mitochondrial membrane phospholipids loss rate between myocardium and skeletal muscle in VMC mice. The correlation of mitochondrial membrane phospholipids normal localization between myocardium and skeletal muscle in VMC mice was described in part b, R = 0.959, p<0.001; the correlation of partial loss of mitochondrial membrane phospholipids was described in part c, R = 0.886, p = 0.03; the correlation of most or complete loss was described in part d, R = 0.951, p = 0.001.

MtDNA^3867^ deletion in skeletal muscle existed in both virus infected and control groups. At day 3, 11, and 24 after the virus infection, mtDNA^3867^ deletion rate significantly increased compared with control group (P<0.05). The mtDNA^3867^ deletion rate at day 11 was significantly higher than that of day 3, and tended to improve at day 24. But it was not statistically different compared with day 11, and still significantly higher than that of day 3 (P<0.05) (Table 3). Spearman correlation analysis showed the deletion rate of mtDNA^3867^ in myocardium was also consistent with that in skeletal muscle with the r value of 0.981 (Fig. 3a).

#### Correlation analysis of cardiac function and myocardial mtDNA^3867^ deletion rate in VMC mice

At day 3 after the virus infection, −dp/dt_max_, which reflects left ventricular diastolic function, was significantly increased in VMC mice (P<0.05). At day 11, the heart functions were further impaired in both systolic and diastolic functions in the virus infected groups, accompanied with a significant reduction in blood pressure. At day 24, the heart functions were improved compared with day 11, but still significantly worse than the control group (Table 3). Spearman correlation analysis showed that there were significantly negative correlations between myocardial mtDNA^3867^ deletion rate and LVPSP (r = −0.66, P<0.05), and between myocardial mtDNA^3867^ deletion rate and +dp/dt_max_ (r = −0.79, P<0.05), while there was significantly positive correlation between myocardial mtDNA^3867^ deletion rate and −dp/dt_max_ (r = 0.80, P<0.05).

### The loss of cardiac mitochondrial membrane phospholipids in VMD patient and animal model

#### The loss of mitochondrial membrane phospholipids in lymphocytes of VMD patients

**Figure 4:**
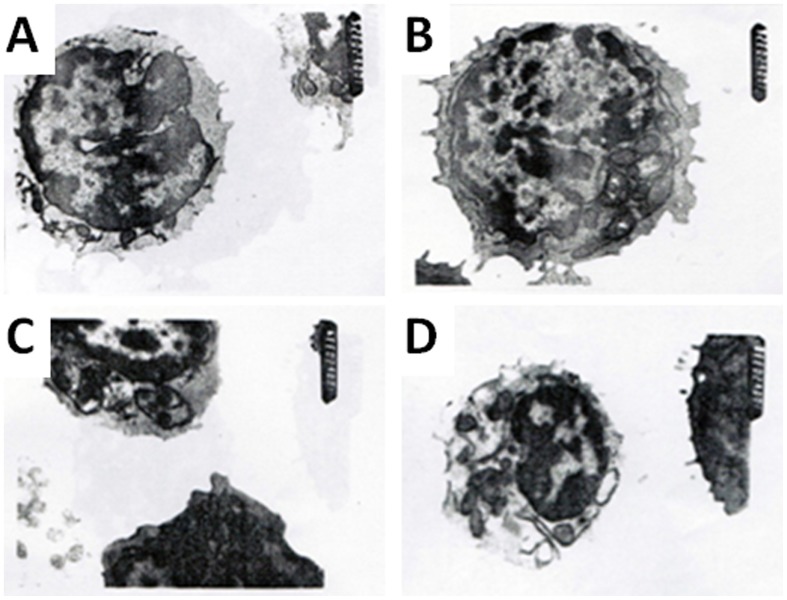
The structure of lymphocytes in control group (a) and VMD patients showing swelling of mitochondria and deletion mitochondrial membrane phospholipids (b, c, d). (magnification 10,000X).

The loss of mitochondrial membrane phospholipids in lymphocytes was found in both VMC and DCM patients (Fig. 4). The loss rate of the mitochondrial membrane phospholipids in lymphocytes was significantly higher in VMC patients compared with normal subjects, while it was further increased in DCM patients (Table 4).

**Table 4:** The loss rate of mitochondrial membrane phospholipids in lymphocytes of VMD patients.

group	n	Normal localization (%)	Partial loss (%)	Most/complete loss (%)
controls	23	88.7±5.1	8.0±4.2	3.3±1.8
VMC	50	68.5±4.3*	20.0±3.4*	11.9±4.7*
DCM	33	42.1±8.5* ^#^	33.2±4.1* ^#^	24.3±5.6* ^#^

*P<0.05 vs. controls; ^#^P<0.01 vs. controls.

#### The loss of cardiac mitochondrial membrane phospholipids in VMC mice

**Figure 5:**
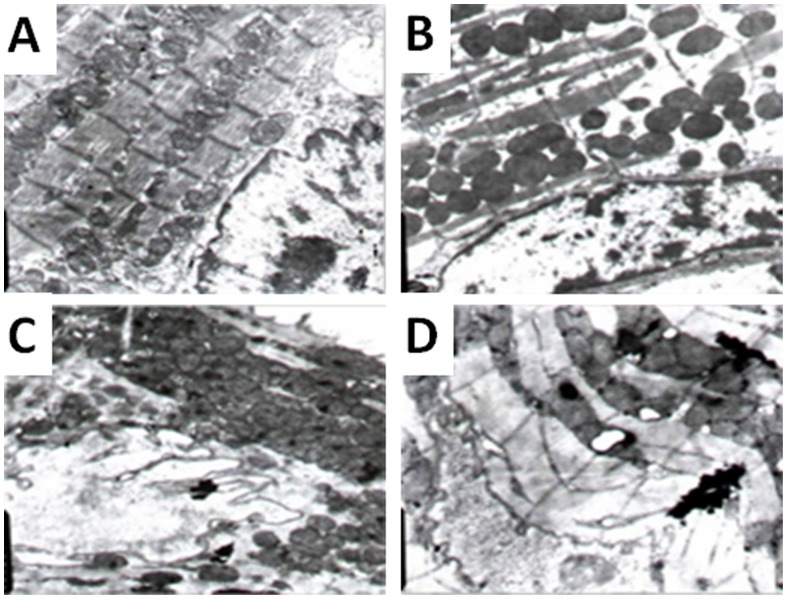
The structure of mitochondria in cardiomyocyte of VMC mice (magnification 10,000X). (**a**) Cardiomyocyte of normal mouse. The mitochondria were circle or elliptical with legible and densely packed cristae. There were also sporadic glycogen granules between the myofibrils. (**b**) Cardiomyocyte of mice 3 days after virus infection. The mitochondria proliferated and swelled, with pyknosis, vesicle and the decreased matrix; glycogen granule decreased or disappeared. (**c**) Cardiomyocyte of mice 11 days after virus infection. The mitochondria proliferated and swelled, with the fusion of mitochondrial membrane and merged to huge mitochondria; glycogen granule decreased; inhomogeneous myofibril dissolved, and the sarcomere disappeared. (**d**) Cardiomyocyte of mice 24 days after virus infection. The mitochondria significantly proliferated and swelled, the cristae became vague; glycogen granule decreased, and sarcoplasmic reticulum dilated.

Results from transmission electron microscope showed the loss of the cardiac mitochondrial membrane phospholipids after the infection of CVB3 (Fig. 5). At day 3 after infection, the loss of mitochondrial membrane phospholipids was different from that in controls, but showed no statistical difference (*P*>0.05). At day 11 after infection, the percentage of normal localization of mitochondrial membrane phospholipids was decreased, whereas the percentage of partial loss or most/complete loss of mitochondrial membrane phospholipids localization significantly increased (*P*<0.05). At day 24 after infection, the percentage of complete/most complete loss of mitochondrial membrane phospholipids was increased and the percentage of partial loss or normal localization of mitochondrial membrane phospholipids dropped, but was still significantly different from that at day 3 after infection (P<0.05) and not different from that at the day 11 after infection (*P*>0.05) (Table 5).

**Table 5:** The loss rate of mitochondrial membrane phospholipids in myocardium and skeletal muscle of VMC mice (mean ± SEM).

		myocardium			skeletal muscle		
Viral infectiondays	n	Normal localization(%)	Partial loss(%)	Most/complete loss(%)	Normal localization(%)	Partial loss(%)	Most/complete loss(%)
controls	3	88.7±0.3	6.5±0.6	4.8±0.4	85.1±3.2	9.9±2.5	5.0±0.7
3	3	80.0±1.4	11.3±1.4	8.3±1.9	80.4±2.5	11.6±1.0	8.0±2.7
11	3	42.9±0.1*	16.5±0.7*	40.6±0.6*	67.4±1.8*	20.7±1.7*	11.9±0.7*
24	3	52.0±0.6* ^#^	16.2±0.5*	31.9±0.1* ^#^	70.3±1.4*	19.0±0.5*	11.0±2.3*

^*^P<0.05 vs. controls; ^#^P<0.05 vs. 3d after viral infection.

#### The loss of mitochondrial membrane phospholipids in skeletal muscle and its correlation with that in myocardium in VMC mouse

**Figure 6:**
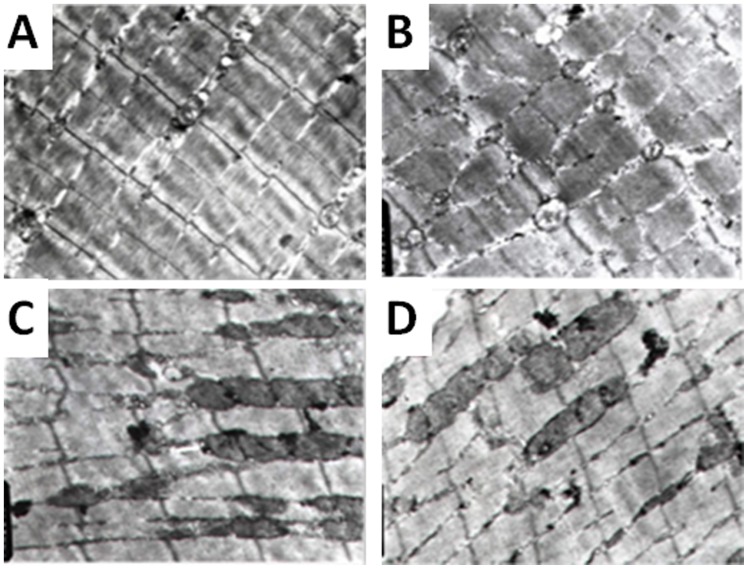
The structure of mitochondria in skeletal muscle of VMC mice (magnification 10,000×). (**a**) Skeletal muscle of normal mouse. The mitochondria were circle or elliptical with legible and densely packed cristae. There were also abundant glycogen granule, legible sarcoplasmic reticulum and T tube near Z line. (**b**) Skeletal muscle in mice 3 days after virus infection. The mitochondria swelled, the number of mitochondrial cristae decreased or disappeared, sarcoplasmic reticulum dilated, and glycogen granule decreased. (**c**) Skeletal muscle in mice 11 days after virus infection. The mitochondria proliferated and swelled; glycogen granule decreased. (**d**) Skeletal muscle in mice 24 days after virus infection. The mitochondria proliferated and swelled; sarcoplasmic reticulum dilated.

The structure of mitochondria in skeletal muscle changed due to virus infection (Fig. 6). At day 3 after infection with CVB3, the loss of mitochondrial membrane phospholipids was different from that of controls, but showed no statistical difference (P>0.05). At day 11 after infection, the loss of mitochondrial membrane phospholipids was significantly higher than that in control group (P<0.05). This difference tended to be attenuated at day 24, but was still significantly higher than that at day 3 (P<0.05) and not different from that at the day 11 after infection (Table 5). Spearman correlation analysis showed the consistence of the impairments of mitochondrial membrane phospholipids in myocardium and skeletal muscle. There was significant correlation between them for mitochondrial membrane phospholipids normal localization, partial loss and most/complete loss, with the correlation coefficients of r = 0.959 (P<0.05), r = 0.886 (P<0.05), and r = 0.951 (P<0.05), respectively (Fig. 3).

## Discussion

Mitochondrion is the most important place for energy metabolism and cellular metabolic network in the cells [7]. MtDNA is responsible for the encoding of 13 kinds of peptides for oxidative phosphorylation (OXPHOS) [9], [24]. Due to the lack of the protection of histidine and effective repair system, mtDNA is easily to be damaged and is characterized with high frequently mutation [25], [26]. There is a strict positioning for respiratory chain enzymes in the mitochondrial inner membrane. Phospholipids is the main component of biomembranes, and provides the important microenvironment for the normal function of mitochondrial oxidative phosphorylation [27]. In cardiomyocytes, mitochondria occupy 35–40% of its volume [28]. The abnormal mitochondrial structure and function is the important molecular mechanism for the impairment of myocardial energy metabolism [12], [29]. In this study, we have determined the effects of mitochondrial damage in myocardial injury of viral cardiomyopathy by studying the mtDNA deletion and the phospholipids membrane damage.

Results from this study have shown that the deletion rate of mtDNA^4977^ and mtDNA^7436^, two common mtDNA deletion types in myocardium, were significantly higher in VMD patients than that in the normal subjects, while it was higher in DCM patients than that in the VMC patients. These results indicate the association of mtDNA deletion and the development of VMC, and that mtDNA deletion might be an important mechanism for the development DCM from VMC. To further study the roles of mtDNA deletions in myocardial injury, we also established a mouse model of viral myocarditis to determine the myocardial mtDNA deletion at various time points after viral infection. The results show that myocardial mtDNA^3867^ deletion appeared as early as day 3 after the viral infection, and was getting worse over the period of the experiment until the late time of the experiment. Interestingly, the cardiac dysfunction after viral infection was consistent with the changes of mtDNA deletion. There was significant correlation between mtDNA deletion rate and the impairment of heart function. These results strongly suggest that mtDNA deletions may be the important pathophysiological mechanisms of myocardial injury and the subsequent cardiac dysfunction in VMD patients.

The main components of biomembrane are lipids and proteins. The phospholipids are one of the important molecules that form the skeleton of biomembrane. The injury of cell membrane phospholipids is the initial manifestation of the cellular structure changes [16]. In the mice with viral myocarditis, the myocardial mitochondrial membrane injury was correlated with mtDNA deletion. The loss of mitochondrial membrane phospholipids and mislocation is associated with the incidence of VMD, and the damage might be irreversible in viral myocarditis without intervention.

As a place for complete oxidative decomposition of nutrients and providing the energy for the cells, mitochondria are essential for the maintenance of cell structure and function [24]. Some of the respiratory chain enzyme protein complexes distribute in the mitochondrial membrane. Electron is transferred during oxidation through the respiratory chain complexes [24]. In this study, we detected mtDNA deletion and mitochondrial membrane damage in the myocardium in both VMD patients and animal models, suggesting that mitochondrial damage may be an important pathophysiological mechanism of myocardial injury in VMD. The mechanisms of the mitochondrial membrane phospholipids and DNA damage in VMD might be due to the excess oxygen free radicals, Ca^2+^ overload, and depletion of intracellular antioxidant enzymes or their activities decreased [30]–[34]. These changes can activate phospholipase to increase phospholipids degradation and the accumulation of the degradation products (lysophospholipids, free fatty acids, etc.), and increase the membrane vulnerability as well [32]. Membrane phospholipids damage will inevitably lead to the dysfunction of respiratory enzyme, protein, receptors in the membranes, and affect the one-way transmission of electron flow [35]. MtDNA in the cells is in an exposed state, lack of histidine protection and effective repair system, which is easy to be damaged and frequently mutated, so it has the high sensitivity to the oxidative stress injury [28]. MtDNA encodes the multi-peptide for oxidative phosphorylation, and the mtDNA deletion can affect oxidative phosphorylation proteins encoding leading to the oxidative phosphorylation abnormalities [12], [36], [37]. This study examined two types of mtDNA deletion. The opportunities of other mtDNA damages including large fragment deletions, mutations, insertion, etc, may also exist in VMD patients. Membrane phospholipids and mtDNA damage result in the loss of mitochondrial oxidative phosphorylation coupling, decreased myocardial ATP synthesis and the abnormalities of energy use, conversion and reserve [38], [39]. When these changes reach a certain level and ATP generation is less than the minimum threshold required for cellular energy metabolism, “power hungry” will appear which is involved in the heart enlargement and heart failure. Nevertheless, heart failure can worsen the abnormalities of myocardial energy metabolism, thus creating a vicious cycle which eventually induces irreversible myocardial injury [10], [11], [40], [41].

For a long time, the difficulties and side effects in myocardial biopsy have prevented the research and the deep and accurate understanding of the pathophysiological and molecular mechanisms of the disease of cardiomyopathy. One of the aims of the present study was to determine the correlation of mitochondrial damage in myocardium with that in peripheral lymphocytes and skeletal muscles, in order to get a peripheral “window” which reflects myocardial injury. We found that the mitochondrial abnormalities in skeletal muscle occurred simultaneously with that in the heart after virus infection in mice. The membrane phospholipids loss and mtDNA deletion occurred at the early time of viral infection when the heart functions were still normal. These damages were getting worse over the time of infection while the heart functions were impaired. These results demonstrate that mitochondria in skeletal muscle can also be damaged in VMC mouse and there is a good correlation for the mitochondrial membrane phospholipids abnormalities and DNA deletion in skeletal muscle and myocardium. Similar changes have also been found in the VMD patients. Lymphocytic mtDNA deletion and the loss of mitochondrial membrane phospholipids have been found in both VMC and DCM patients. The incidence in DCM group was significantly higher than that in VMC group, while both groups were higher than the control subjects. There were significant correlations for both mtDNA deletion and the loss of mitochondrial membrane phospholipids between lymphocytes and myocardium. These results indicate that the skeletal muscle and lymphocytes might be a peripheral “window” reflecting myocardial mitochondrial damages. Further studies are needed in both animal models and patients to explore the mitochondrial function changes due to mtDNA deletion and the loss of mitochondrial membrane phospholipids in skeletal muscle, lymphocytes, and myocardium.

### Limitations in this study

We also realized the limitations of this study. First, due to the difficulty and limitation of getting human samples, we were not able to get the blood samples from the 12 cases of healthy accidental death, so we collected blood samples from other 23 healthy blood donors as the controls. This limited the correlation assay between blood and myocardial samples. Second, mtDNA4977 and mtDNA7436 are very common mtDNA deletion types in the heart, which may induce the abnormality of oxidative phosphorylation and energy metabolism in the heart, and be involved in the development of DCM [21]. However, we could not exclude the possibility that other mtDNA deletion types may also exist and participate in the pathogenesis of DCM. Third, for patients with viral myocarditis, we have determined the blood coxsackievirus B-IgM and the results were positive for all of the 50 patients. While all the DCM patients included in this study had the history of viral myocarditis, the pathological study for viral infection was not performed again during this study in these DCM patients. Fourth, we also realized that mtDNA deletion exist in various types of diseases [42]. This study demonstrates correlations of mitochondrial damage in the myocardium, skeletal muscle and lymphocytes in VMD. However, whether these relationships also exist in non-viral infectious diseases is still unclear and need further investigations.

In summary, the abnormalities of mitochondrial membrane structure and mtDNA have been reported in dilated cardiomyopathy, cardiac conduction defects, sudden death, ischemic heart disease, and degenerative heart disease [43]. This study determined the correlation of mitochondrial damage and the development of VMD in both VMD patients and animal models, and found that mitochondrial damage may be an important pathophysiological mechanism leading to myocardial injury and cardiac dysfunction. The mechanisms of mitochondrial damage and the exact roles in the myocardial injury need to be further studied. We also studied the correlation of mitochondrial damage in myocardium and skeletal muscle and lymphocytes and found that the mitochondrial damage in the skeletal muscle and lymphocytes might reflect a “window” of myocardial mitochondrial damage, which will help us to further study and understand the pathogenesis, pathophysiology and molecular mechanisms of VMD.
